# Using measures of reading time regularity (RTR) to quantify eye movement dynamics, and how they are shaped by linguistic information

**DOI:** 10.1167/jov.22.6.9

**Published:** 2022-05-25

**Authors:** Monika Tschense, Sebastian Wallot

**Affiliations:** Institute of Psychology, Leuphana University of Lüneburg, Lüneburg, Germany; Research Group Neurocognition of Music and Language, Max Planck Institute for Empirical Aesthetics, Frankfurt am Main, Germany; Department of Language and Literature, Max Planck Institute for Empirical Aesthetics, Frankfurt am Main, Germany; Institute of Psychology, Leuphana University of Lüneburg, Lüneburg, Germany; Department of Language and Literature, Max Planck Institute for Empirical Aesthetics, Frankfurt am Main, Germany

**Keywords:** reading time regularity, information processing, recurrence quantification analysis, sample entropy analysis, text reading

## Abstract

In this article, we present the concept of reading time regularity (RTR) as a measure to capture reading process dynamics. The first study is concerned with examining one of the assumptions of RTR, namely, that process measures of reading, such as eye movement fluctuations and fixation durations, exhibit higher regularity when contingent on sequentially structured information, such as texts. To test this, eye movements of 26 German native speakers were recorded during reading-unrelated and reading-related tasks. To analyze the data, we used recurrence quantification analysis and sample entropy analysis to quantify the degree of temporal structure in time series of gaze steps and fixation durations. The results showed that eye movements become more regular in reading compared to nonreading conditions. These effects were most prominent when calculated on the basis of gaze step data. In a second study, eye movements of 27 native speakers of German were recorded for five conditions with increasing linguistic information. The results replicate the findings of the first study, verifying that these effects are not due to mere differences in task instructions between conditions. Implications for the concept of RTR and for future studies using these metrics in reading research are discussed.

## Introduction

What guides the reading process? Reading is a complex cognitive process bringing together perceptual-motoric skills, executive functions, memory capacities, and language knowledge (e.g., Rayner & Reichle, 2010). A general assumption all theories and models of reading share is that the reading process is driven by linguistic features of written language, at least to some extent. This is particularly evident for the front-end processes of reading, such as visual word recognition, where lexical features (e.g., word length, word frequency, semantic properties) substantially impact word reading times (Grainger & Jacobs, 1996; Ziegler et al., 2000). Consequently, it is implemented in more encompassing models of eye movements during reading in which lexical features govern fixation durations and saccadic programming (Engbert et al., 2005; Reichle et al., 2009). Moreover, higher-level theories of reading and models of discourse comprehension assume that linguistic features of a text, such as propositional density, situation model dimensions, and syntactic complexity, drive reading times for connected text (Graesser et al., 2004; Kintsch & Keenan, 1973; Zwaan et al., 1995). This is further supported by studies showing that mind-wandering during reading leads to a detachment of eye movement measures from linguistic text features (Faber et al., 2020; Schad et al., 2012). Hence, a basic presumption might be that there is indeed a systematic relationship between linguistic text features and the reading process. Following this line of thought, linguistic features should account for (a large fraction of) the variance of observables of the reading process (e.g., word frequency should unequivocally predict sentence reading times).

However, the coupling between reader performance and linguistic text characteristics strongly varies between individuals (Rayner et al., 2006; Traxler et al., 2012), tasks (Teng et al., 2016; Wallot et al., 2013), and languages (Frost, 2012; Holden & Van Orden, 2002). For example, the effect sizes of word frequency and word length differ substantially between reading tasks presenting isolated words or sentences as compared to reading longer, connected texts. Wallot and colleagues (2014) report smaller effect sizes for connected texts compared to reading tasks that emphasize shorter language segments. Besides, there is evidence that effects of lexical features decrease systematically for reading of connected text (Wallot et al., 2013). Furthermore, such effects can even depend entirely on the order in which reading tasks are performed. As shown by Teng and colleagues (2016), word frequency effects for a lexical decision task disappeared when participants had performed a connected text reading task beforehand, while the frequency effect stayed completely intact when the lexical decision task was performed first.

This variability of results regarding the relationship between text features and measures of the reading process is evident not only across tasks but also across languages (Frost, 2012). So showed Holden and Van Orden (2002) that the strength of the word frequency effect varies rather strongly for different languages. Similarly, reading in many languages has been shown to be quite robust regarding changes in letter order, which has been subsequently described as a core property of reading at the neurophysiological level (Whitney & Cornelissen, 2005). Yet, research shows that changes in letter order pose a great challenge for readers of Hebrew (Velan & Frost, 2007). Taken together, it is clear that text features play an important role in controlling the reading process, but the way they do so is not easy to generalize across reading situations, languages, and readers. This also makes it difficult to build a general theory of the reading process based on text features as its driving factors.

### Reading time regularity

We thus introduce the concept of reading time regularity (RTR) as a general means to assess the influence of (linguistic) information on perceptual-cognitive processes during reading (Wallot, 2014, 2016). From the perspective of RTR, a process that has a high degree of regularity is a process that evolves comparatively stable over time. Such a process is not subject to larger perturbations or dampens them out quickly. Perturbations of the reading process usually result in conjunction with problems of concentration (e.g., mind-wandering: Faber et al., 2020; Schad et al., 2012), comprehension and text difficulty (Rayner et al., 2006), reading skill (Reichle et al., 2013), or surprise or failure of prediction (Booth et al., 2018). This means that a reader does not efficiently continue to read but has to integrate information differently, search for information, or change the situation model (McNerney et al., 2011). Such changes are usually evident in the reading time course as reflected in long reading times, increased variability of reading times, or specific eye movements, such as regressions.

If a reader is skilled, he or she will be able to solve such conflicts quickly and restore comprehension, so that misunderstandings do not increase the probability for additional comprehension problems later in the text. Both the quick resolution of such conflicts, as well as the reduced probability of encountering such conflicts, will reduce the variability of reading process measures, such as word reading times or eye movement fluctuation, and hence increase the temporal structure, the regularity of the process measure in question. Accordingly, regularity can be seen as a marker of skilled and efficient reading.

Or course, the basic input for what is efficient reading or reading problems is the linguistic information present in a text, which can span the whole range of sublexical, lexical, semantic, syntactic, and discourse-level features. As we have laid out above, the problem is that the effects of each of these features is highly variable across task, person, and language when trying to relate specific text features to changes in reading process measures, but observables.

Here, we propose that RTR might offer a solution to the problem of the variability with which linguistic features relate to measures of the reading process. As explained above, a reading process of high regularity captures efficient and skilled reading, and accordingly good or at least sufficient comprehension. In order to draw this conclusion, however, we do not need to relate specific text features to changes in reading process measures, but we can simply make such an inference based on the relative degree of regularity.

This also means that we do not need to make particular assumptions about the effect of particular text features in question, or how several of such features might interact to bring about a particular effect, or why such an effect seems to be strong under some reading conditions but weak under others. We can assume that there is a coupling between the relevant linguistic information in a particular instance of reading and the cognitive-perceptual processes involved in reading, and if that coupling is efficient and functional, this will be marked by a high degree of regularity.

Our proposal rests on the following assumptions:

A1. Any observable that can be used to measure the reading process (e.g., eye movements) is inherently a random variable of sorts.
A2. When this variable is measured in a reading situation, its values become contingent on some properties of the text that are relevant for the reader (e.g., fixations durations become correlated with lexical word properties).
A3. Because texts are inherently hierarchically ordered sequences (e.g., from topic to syntax/word order to lexical—and sublexical—properties), a random variable that becomes contingent on this sequence will exhibit increased order.
A4. Because ability of the reader to couple with a text depends on reading skill and comprehension, efficient coupling implies higher degrees of regularity.

Assumptions A2 and A4 are to some degree restatements of the general assumption shared by all models of reading, namely, that linguistic features co-control the reading process. Importantly, however, in the logic of RTR, linguistic text features are not necessary to describe the coupling between reader and text, but it can be inferred from the degree of regularity of a reading process measure alone.

Statistically, RTR captures the regularity, that is, autocorrelation properties, of process measures. Hence, the degree of RTR of a reading process measure can in principle be calculated by any statistic that captures order of a sequence or time series, such as recurrence quantification analysis (Zbilut & Webber, 1992), or sample entropy analysis (Richman & Moorman, 2000). The fact that RTR is solely based on the values of an observable of the reading process, specifically on their sequential properties, but not particularly on text features, can address the challenges outlined above. This is what distinguishes RTR from other attempts to define cognitive coupling (e.g., Mills et al., 2017). Before summarizing some potential applications of RTR in reading research, we provide a brief description of the regularity measures employed in this study. Further information about the parameter estimation for these measures is provided in the Method section.

#### Measures of regularity

##### Recurrence quantification analysis

Recurrence quantification analysis (RQA) can be used to quantify various dynamic properties of a time series related to the degree structure of its temporal evolution. Effectively, the RQA measures we employ here capture different kinds of autocorrelation in a time series. They capture different aspects of clustering of data points over time, which is how, i.e., individual data points forming larger patterns within a time series. This can be visualized by means of recurrence plots (RPs) based on which several complexity measures can be derived quantifying the density of recurrence points and their line structures (Zbilut & Webber, 1992). Several RQA measures can be extracted from an RP, but we will focus on the most common measures—recurrence rate (RR), determinism rate (DET), average diagonal line length (ADL), and maximum diagonal line length (MDL): The RR refers to the density of recurrence points, providing information about the repetitiveness of individual values or coordinates within a time series. The less stochastic and the more deterministic a process is, the more recurrent points occur in connected trajectories as opposed to single recurrence points. How many recurrent points occur in diagonal lines as opposed to individual repetitions is indicated by DET. The line length can also be extracted, either as ADL or as MDL. While these measures can distinguish different dynamics properties in certain systems (Marwan et al., 2007), in data with a strong stochastic component, such as eye movement fluctuations, they are often highly correlated. Accordingly, we aim to investigate whether all or just some of them make good indicators of regularity.

RQA has been applied to a variety of research areas, but it has also been used to analyze reading times from dyslexics and nonimpaired controls during a naming task (Wijnants et al., 2012), as well as text reading times of children and adults (Wallot et al., 2014). These studies report lower RQA measures for dyslexic reading compared to controls and that higher RQA measures correlated positively with reading speed and comprehension, probably reflecting a more skilled and efficient processing of text. In line with these results, higher values of RR, DET, ADL, and MLD indicate higher regularity according to RTR.

##### Sample entropy analysis

Sample entropy analysis (SampEn) quantifies the degree of predictability of a time series (Richman & Moorman, 2000). It takes into account the number of matching sequences identified within a tolerance band defined by a radius *r*, excluding self-matches. Specifically, SampEn is the average probability that a sequence with length of *m* + 1 data points finds a matching sequence within *r*, given that a match for *m* data points has already been found. Highly periodic, deterministic time series are easily predictable (i.e., if sequences of *m* points repeat, then sequences of *m* + 1 points are also likely to repeat), yielding a *SampEn* = 0. In contrast, a time series that is very noisy yields a *SampEn* > 0.

While sample entropy has been increasingly employed in sport science and motor control research, it has not yet been used to investigate reading data. As a measure of entropy, higher values of SampEn might indicate lower regularity in terms of RTR. However, because RTR is not about entropy per se but about how well patterns of different length are contained within each other, SampEn might behave more like an entropy rate measure (Porta et al., 2001). That is a measure of complexity, and as such, SampEn might in fact be higher during reading compared to baseline conditions with fewer external information to be processed.

#### Potential applications of reading time regularity in reading research

Insofar as some of the measures described above turn out to be a valid metrics for capturing functional coupling of linguistic information and perceptual-cognitive processing, RTR has potential applications for reading research. First of all, RTR might make a suitable measure of reading fluency. While reading fluency is conceived as relatively effortless reading with at least average to good comprehension (O'Brien et al., 2014), it is often operationalized as overall reading speed or reading time components. Here, level of speed is used as a stand-in measure for the reading process, because of the positive correlation between skill and reading speed (Fuchs et al., 2001). However, reading speed during text reading is not always substantially related to comprehension, calling this relationship into question (LeVasseur et al., 2006, 2008; Wallot et al., 2014). Instead of using speed as a key characteristic of the reading process, it can equally be seen as an outcome of reading ability and hence reading fluency instead of being a process per se. So far, this circularity issue constitutes an experimental confound in the presumed positive relationship of reading speed and comprehension, which is difficult to avoid empirically. Moreover, the relationship between reading speed and comprehension is complex: While an increase in reading speed can lead to a decrease in comprehension in a trade-off relationship, it can also lead to increases in comprehension. But speed is also thought to correlate positively with comprehension as a general aspect of reading ability.

Therefore, adding the concept of RTR into an operational definition of reading fluency might be able to resolve this conceptual problem: When RTR is used as a measure for reading process fluency in the sense of an effortful, functional execution of the reading process, speed can be solely treated as an outcome variable—and measures of reading time regularity have shown a predictive link to reading speed and comprehension, as well as capture their trade-off relation very well (Wallot et al., 2014).

Since the calculation of RTR does not depend on specific linguistic text features, it can, in principle, be used as a cross-linguistic measure for the prediction of reading comprehension, irrespective of the particular properties of different writing systems and their consequences for reading.

Prior work using measures of regularity of the reading process has shown that the degree of regularity in reading time data is positively correlated to reading comprehension. Notably, RTR properties reliably predicted text comprehension better than reading speed (O'Brien et al., 2014; O'Brien & Wallot, 2016; Wallot et al., 2014), and preliminary results from an eye tracking study corroborated the power of RTR measures in predicting text comprehension using eye movements over and above standard eye movement features related to comprehension, such as fixation durations, number of fixations, and percentage of regressive eye movements (Wallot et al., 2015).

However, these results were obtained before the formulation of RTR and formed the basis for this concept. No prospective tests of this hypothesis have been performed, and, crucially, none of the assumptions (A1–A4) outlined above have been prospectively tested. Hence, the goal of the current article is to test and investigate the foundational measurement assumptions of RTR, particularly A2 and A3, regarding the basic effect of (linguistic) information on process measures—time series of eye movement records—on measures that capture the regularity of such time series. We will return to the discussion of applications of RTR in reading research at the end of the discussion section.

## Experiment 1

**Figure 1.**
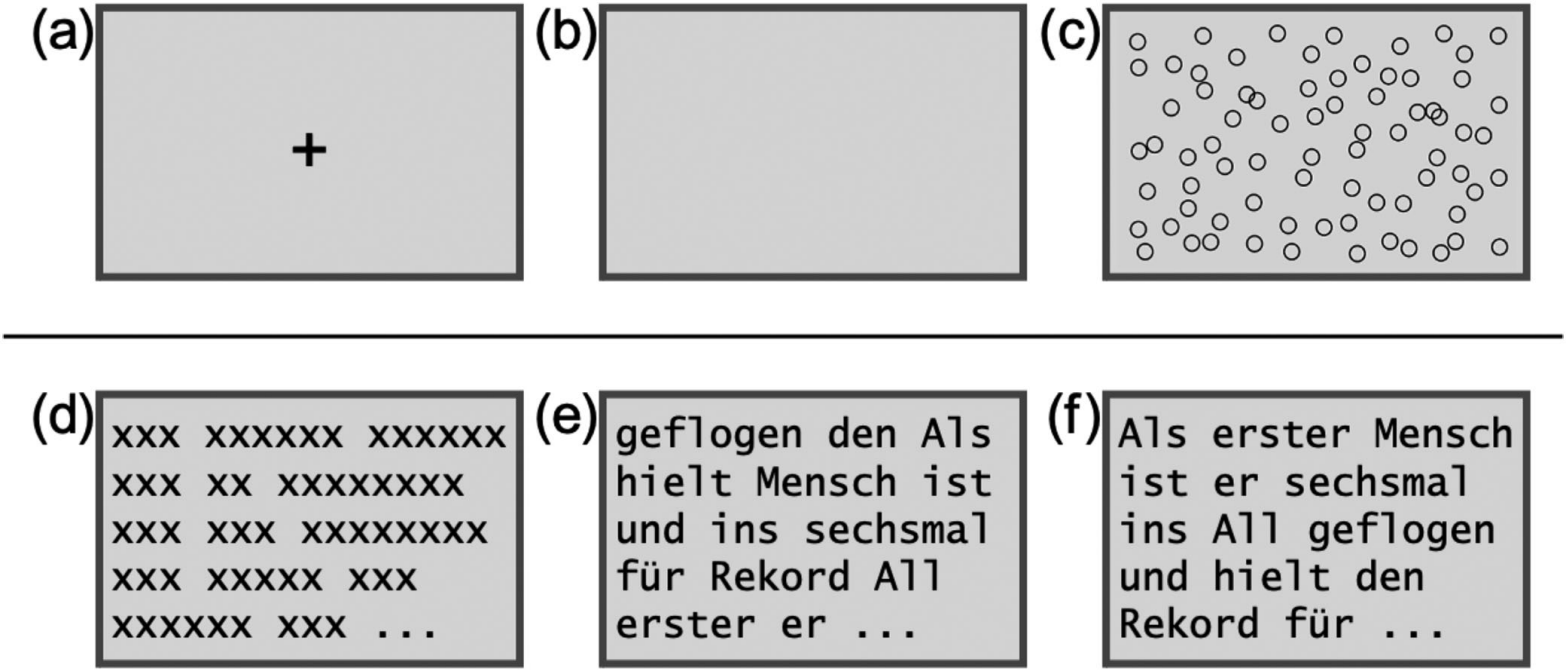
Schematic examples for the experimental conditions. The top panel illustrates the baseline conditions with (a) fixation cross, (b) blank screen, and (c) circles. The bottom panel shows the reading conditions with (d) text grid, (e) shuffled text, and (f) normal text.

In order to test one of the basic assumptions of RTR, namely that the presence of external (linguistic) information leads to an increase in process regularity, we constructed an eye movements experiment. We included six conditions: Three contained little to no visual information, two contained information associated with reading, and one condition contained proper text. Figure 1 illustrates the conditions. Participants’ eye movements were subjected to RQA, FA, and SampEn in order to quantify the degree of regularity of eye movements in each of these conditions.

### Hypotheses

Drawing on the concept of RTR, it is hypothesized that the presence of external linguistic information (see Figures 1d–f) leads to increases in regularity compared to control conditions that do not contain such information (see Figures 1a–c). This is expected because the coupling between cognitive processing and the sequential structure of that information leads eye movement dynamics to become more regular. This hypothesis is tested using gaze step (Stephen & Mirman, 2010). The gaze step is the spatial distance between two position measures of the raw eye movement record (see Method section for details on calculating gaze step). This is done because some of the baseline conditions, particularly the fixation cross and blank screen conditions, exhibit drift-like behavior and are not well parsable into fixation durations (Yarbus, 1967/2013) since fixations are largely absent in the respective time series.

In an exploratory part, we will evaluate to what extent the reading conditions (see Figures 1d–f) can be distinguished from one another by means of the described regularity measures. Since normal text provides the maximal degree of linguistic information possible during reading, we predict the text condition to lead to increased regularity in eye movement dynamics compared to text grids and shuffled texts. However, it is currently unknown which of the regularity measures described above capture these differences best—or at all. Analyses will be based on both gaze step data as well as time series of fixation durations extracted for the three reading conditions. A more general aim of this study is also to test several regularity indicators (recurrence and entropy measures) that might be principally suitable for the operationalization of RTR with regard to their validity and sensitivity to distinguish between conditions exhibiting differences regarding their degree of external (linguistic) information.

### Method

#### Participants

Twenty-six native speakers of German with normal or corrected-to-normal vision participated in the study and received a compensation of 15€. One participant terminated the experiment before completion and was therefore discarded from any analysis. Due to technical problems during calibration procedure and data recording, two other participants had to be excluded. Furthermore, data of a fourth participant was excluded due to excessive artifacts and blinks. Thus, the final sample consisted of 22 participants (15 female) with a mean age of 27.63 years (*SD* = 9.59). See Appendix A for further information about the participants. Prior to the experiment, written informed consent was obtained from all participants. The study was approved by the Ethics Council of the Max Planck Society and followed the ethical principles of the Declaration of Helsinki.

#### Stimuli

The experiment was composed of six conditions, including three conditions unrelated to reading, another two conditions reflecting certain aspects of reading, and one condition consisting of normal text reading (see Figure 1). For the reading-unrelated conditions (baseline conditions), participants were shown (a) a static fixation cross in the middle of the screen, (b) a blank screen, or (c) a screen filled with circles. For the circle condition, 500 circles with black outline at a size of 10 px were randomly distributed on the screen, and a total of seven circle patterns were created.

The other three conditions (reading conditions) consisted of (d) text grids, (e) shuffled texts, or (f) actual newspaper texts. Reading conditions were based on articles from the German daily newspaper *Die Welt* published in January 2018. Chosen articles consisted of 150 to 200 words and did not concern highly divisive topics. For seven newspaper sections (Economics, Feuilleton, Finances, Politics, Science, Society, Sports), two articles each were selected and randomly assigned to one of two lists. Some key descriptive text characteristics are summarized in Table 1. See Appendix B for all characteristics collected.

**Table 1.** Text characteristics of the selected newspaper articles for List A and List B. *Notes*: Number of syllables, type frequency, and annotated type frequency were obtained from dlexDB (Heister et al., 2011). Given frequency values are logarithmized.

						Type frequency		Annotated type frequency	
List	Section	Words	Sentences	Words per sentence	Syllables per word	*M*	*SD*	*M*	*SD*
List A	Economics	180	10	18.00	2.08	4.18	1.54	4.06	1.54
	Feuilleton	195	10	19.50	1.92	3.98	1.96	3.87	1.96
	Finances	194	11	17.64	2.30	3.79	1.92	3.66	1.92
	Politics	177	11	16.09	2.29	4.03	1.93	3.81	1.93
	Science	157	9	17.44	2.18	3.80	1.85	3.72	1.85
	Society	201	15	13.40	1.98	4.27	1.57	4.11	1.57
	Sports	189	12	15.75	2.06	3.93	2.02	3.74	2.02
	Overall	184.71	11.14	16.83	2.12	4.00	0.17	3.85	0.17
List B	Economics	197	14	14.07	2.25	3.98	1.94	3.91	1.94
	Feuilleton	162	10	16.20	2.28	3.87	1.92	3.75	1.92
	Finances	197	12	16.42	2.21	3.68	2.06	3.52	2.06
	Politics	187	9	20.78	2.10	4.08	1.74	3.94	1.74
	Science	179	10	17.90	2.04	4.01	1.91	3.82	1.91
	Society	189	14	13.50	2.06	4.09	1.79	3.90	1.79
	Sports	158	7	22.57	2.23	3.88	1.97	3.76	1.97
	Overall	181.29	10.86	17.35	2.17	3.94	0.14	3.80	0.14

For conditions (d) and (e), all special characters within a text were removed and all content-dependent or infrequent abbreviations were fully spelled out. Subsequently, every letter got replaced by “x”, resulting in grid-like structures for condition (d). While text grids reveal certain surface characteristics (e.g., word length), they prohibit any semantic access. For condition (e), a random sequence of words was generated by shuffling the text of the newspaper articles. Thus, a coherent, in-depth processing beyond the individual word semantics was not possible.

#### Procedure

The study was carried out in a soundproof both with dimmed light. Participants were seated 70 cm in front of an LCD monitor (size: 24 in., refresh rate: 144 Hz, resolution: 1920 × 1080 px). Their head was supported by a head and chin rest to obtain high tracking accuracy. An EyeLink 1000 (SR-Research, Ottawa, Ontario, Canada) was used for monocular data recording of the left eye at a sampling rate of 1000 Hz. Visual stimuli were presented in white on a black background. Fixation cross was presented with 1° visual angle, circle diameter was 0.3° visual angle, and letter width was 0.5° visual angle.

The experiment was conducted in one session that took approximately 90 minutes, depending on participants’ individual reading speed. Halfway through the experiment, participants were allowed to take a short break. At the beginning of each half of the experiment, a 12-point calibration with random sequence was performed, followed by a validation of the measured points. A questionnaire succeeded the experiment to gather demographic information.

Participants were randomly assigned to one of two stimulus lists that differed in terms of newspaper articles: Either they were shown Set A, consisting of seven newspaper articles as coherent texts, and or Set B, including the other seven newspaper articles as shuffled texts and text grids, or vice versa. However, texts were selected so that each set contained one article from each of the seven sections of the newspaper (see stimuli above). Participants were presented with seven trials per condition, resulting in a total of 42 trials per participant. The sequence of trials was fully randomized for each participant.

While participants were asked to fixate the fixation cross for (a), they were allowed to look freely onto the screen for (b) and (c). However, participants were instructed that their gaze should remain on the screen for the whole time of the trial. For the baseline trials, a fixed presentation duration of 60 seconds was chosen, which roughly corresponds to a reading speed of 200 words per minute (e.g., Rayner et al., 2016; Trauzettel-Klosinski & Dietz, 2012) and thus to the approximate duration of the reading conditions. Each item of the reading conditions was proceeded by a fixation cross (0.5 seconds) that marked the beginning of the first word (grid). Participants were then instructed to fixate each word grid (d) or read every word (e) from top left to bottom right. Regarding the text condition (f), participants were asked to read the newspaper article in a normal manner and at a comfortable pace. There was no time limit for the reading trials, allowing participants to proceeded in a self-paced manner.

#### Data analysis

The data of the study are available here: https://osf.io/5eysw/.

##### Preprocessing

Blinks were detected by an algorithm based on pupillometry noise (Hershman et al., 2018) and removed from the data. When more than 10% of data points of a trial were defective, the entire trial was excluded from further analysis. In addition, participants with fewer than three remaining trials per condition were excluded from further analysis. This procedure resulted in the exclusion of one participant and a total of 25 out of 924 trials (2.71%).

As the dependent variable, gaze steps were computed by differencing the raw two-dimensional position data (Stephen & Mirman, 2010). Gaze steps are thus based on consecutive samples of gaze data and not on fixation positions. For instance, the following gaze positions were recorded: [*x*_1_ = 10, *y*_1_ = 15] and [*x*_2_ = 12, *y*_2_ = 14]. Here, the gaze step can be calculated as

$$\begin{array}{ccc} & & \sqrt{{\left(x_{2}-x_{1}\right)}^{2}+{\left(y_{2}-y_{1}\right)}^{2}}\\ & & \;=\sqrt{{\left(12-10\right)}^{2}+{\left(14-15\right)}^{2}}=2.24. \end{array}$$

This way, series of position recordings were transformed into series of gaze steps for each trial. Extreme values deviating more than 10 *SD* from the mean of a time series were discarded. Furthermore, fixation durations for trials of the reading conditions were extracted from the data using the Microsaccade Toolbox for R (Engbert et al., 2015). We specified 6 as the minimal number of samples constituting a saccade and used the default velocity factor of 5. Subsequently, both measures were subjected to RQA (Zbilut & Webber, 1992) using the crqa package for R (Coco et al., 2021). Furthermore, SampEn was calculated using a custom script in MATLAB (v2018b). RQA and SampEn were calculated per trial using the parameters described in the following sections.

##### RQA

In order to run RQA, a delay parameter *τ* and an embedding parameter *D* had to be estimated by computing the average mutual information and false nearest neighbor functions. The *z*-scored data were then subjected to RQA. Following suggestions from Wallot (2017), a threshold parameter *T* was chosen by an iterative procedure, resulting in a mean RR between 5% and 10% across the whole sample of trials and participants. For gaze step data, the parameters were as follows: *τ* = 7, *D* = 7, and *T* = 0.3 (*M*_RR_ = 7.50, *SD*_RR_ = 5.93). For fixation duration data, the following parameters were chosen: *τ* = 2, *D* = 3, and *T* = 0.8 (*M*_RR_ = 7.57, *SD*_RR_ = 4.21). Due to computational limits, RQA for gaze step data was performed in a windowed manner with 10,000 data points at a time in steps of 5,000 data points and then averaged per trial. A tutorial introduction to recurrence quantification analysis is provided by Wallot (2017).

*Sample* *entropy* *analysis (SampEn)*. The basis for computing SampEn is calculating the number of matching sequences of some length *m* and *m* + 1 within a tolerance band defined by a radius *r*. Both parameters need to be set for analysis (Richman & Moorman, 2000). Here, we determined the length of the template *m* and the size of the tolerance region *r* following an approach proposed by Ramdani and colleagues (2009). Regarding our data, we chose *m* = 1 and *r* = 3.0 for gaze step data and *m* = 1 and *r* = 3 for fixation durations. A tutorial introduction to sample entropy analysis is provided by Kuznetsov and colleagues (2013).

*Inferential* *statistics*. As can be inferred from hypotheses and design, this study is organized in two parts: a confirmatory part based on gaze step data and an exploratory one based on both gaze step data and fixation durations. Regarding the confirmatory part, we were primarily interested in differences between baseline conditions and reading conditions. Consequently, the respective experimental conditions were subsumed into one overarching factor, with “baseline” and “reading” being the factor levels. However, since the underlying conditions differ from one another, they still were included as a random factor within the multilevel models that were run. For the exploratory part, the individual conditions came into focus, especially the relationship between text grids, shuffled text and normal text. Hence, these conditions were then treated as one fixed factor with three levels in the multilevel models.

The different RQA measures and SampEn, which we obtained for every trial per participant and condition, were subjected to linear mixed-effects models to account for their nested structure (Richter, 2006). The models were set up in RStudio (v1.2.1335) using the lme4 package (v1.1-23) and tested for statistical significance using the lmerTest package (3.1-2). Our model used the following general form:

$$y_{mi}=y_{00}+y_{01}CONT_{mi}+\upsilon_{0i}+{ɛ}_{mi},ɛ∼N\left(0,\sigma^{2}\right)$$

Here, *y*_00_ is the fixed intercept, *y*_01_*CONT_mi_* is the fixed effect of the contrast of interest, $\upsilon_{0i}$ is the random intercept for participants, and ε_*mi*_ is the error term. Some of the models also include a random intercept for condition $\upsilon_{1i}$ whenever $\upsilon_{1i}$ contributed significantly to the model.

#### Results

While the baseline trials were presented with a fixed duration of 60 seconds, the duration of the reading trials depended on individual viewing times. On average, participants spent 82.28 seconds (*SD* = 38.75) on text grids, 65.36 seconds (*SD* = 21.38) on shuffled texts, and 53.23 seconds (*SD* = 10.69) on texts. Descriptive statistics for each dependent variable are provided in Table 2 for gaze step and in Table 3 for fixation duration data. Especially for gaze step data, RQA measures and SampEn showed high intercorrelations (see Table 4), reflecting that they all capture the concept of regularity as was expected. However, these measures are less intercorrelated for fixation durations (see Table 5).

**Table 2.** Descriptive statistics for dependent variables based on gaze step data.

		RR		DET		ADL		MDL		SampEn	
Condition	Number of trials	*M*	*SD*	*M*	*SD*	*M*	*SD*	*M*	*SD*	*M*	*SD*
(a) Fixation cross	150	3.34	3.27	23.84	18.04	2.13	1.64	68.36	72.72	0.034	0.010
(b) Blank screen	145	4.19	3.26	30.51	17.14	2.69	1.32	81.48	72.24	0.035	0.012
(c) Screen with circles	149	5.01	3.89	37.61	21.98	2.92	1.38	95.92	80.40	0.047	0.014
(d) Text grid	153	10.43	6.56	64.13	24.91	6.19	5.70	204.31	131.20	0.065	0.012
(e) Shuffled text	151	10.78	5.97	69.91	23.12	6.98	6.07	208.41	119.95	0.067	0.009
(f) Normal text	151	11.01	5.43	71.30	20.78	8.40	6.77	215.38	107.41	0.073	0.008
(a–c) Baseline conditions	444	4.18	3.55	30.64	19.96	2.58	1.49	81.89	75.91	0.039	0.013
(d–f) Reading conditions	455	10.74	6.00	68.43	23.17	7.19	6.25	209.34	119.79	0.068	0.010

**Table 3.** Descriptive statistics for dependent variables based on fixation duration data.

	Number of fixations per trial		Fixation duration (ms)		RR		DET		ADL		MDL		SampEn	
Condition	*M*	*SD*	*M*	*SD*	*M*	*SD*	*M*	*SD*	*M*	*SD*	*M*	*SD*	*M*	*SD*
(d) Text grid	253.14	102.09	271.55	37.68	8.15	5.51	20.27	7.01	4.37	1.25	249.14	102.09	0.041	0.007
(e) Shuffled text	251.38	85.53	216.87	19.31	7.20	3.42	17.98	5.02	4.49	0.95	247.38	85.53	0.042	0.009
(f) Normal text	213.48	39.02	205.30	15.63	7.36	3.38	18.13	4.28	4.83	1.40	209.48	39.02	0.041	0.007

**Table 4.** Correlation matrix for dependent variables based on gaze step data. *Notes*: Pearson's *r* correlation coefficients. All coefficients are significant at the *p* < 0.001 level.

Characteristic	RR	DET	ADL	MDL	SampEn
RR	—	0.94	0.79	0.95	0.49
DET	0.94	—	0.68	0.92	0.63
ADL	0.79	0.68	—	0.77	0.40
MDL	0.95	0.92	0.77	—	0.50
SampEn	0.49	0.63	0.40	0.50	—

**Table 5.** Correlation matrix for dependent variables based on fixation duration data. *Notes*: Pearson's *r* correlation coefficients. * *p* < 0.05, *** *p* < 0.001.

Measure	Fixation duration	RR	DET	ADL	MDL	SampEn
Fixation duration	—	0.01	0.00	–0.21 ***	0.33 ***	–0.07
RR	0.01	—	0.85 ***	–0.23 ***	–0.10 *	0.03
DET	0.00	0.85 ***	—	–0.08	–0.29 ***	0.06
ADL	–0.21***	–0.23 ***	–0.08	—	–0.41 ***	0.05
MDL	0.33 ***	–0.10 *	–0.29 ***	–0.41 ***	—	–0.05
SampEn	–0.07	0.03	0.06	0.05	–0.05	—

*Confirmatory analysis: baseline vs. reading conditions*. To test for differences between baseline conditions and reading conditions, linear mixed-effects models were constructed separately for each RQA measure and SampEn. Condition type (baseline vs. reading) was set as categorical fixed effect, and participant and condition were included as random intercepts.

All RQA measures as well as SampEn were affected by condition type (RR: χ^2^(1) = 20.22, ***p < 0.001; DET: χ^2^(1) = 16.27, ***p < 0.001; ADL: χ^2^(1) = 13.70, ***p < 0.001; MDL: χ^2^(1) = 21.57, ***p < 0.001; SampEn: χ^2^(1) = 13.88, ***p < 0.001). All dependent measures distinguished significantly between the two condition types: Compared to reading conditions, baseline conditions exhibit smaller RR, DET, ADL, and MDL, as well as smaller SampEn. Fixed effects for all measures are summarized in Table 6.

**Table 6.** RQA measures and SampEn for gaze step data: Fixed effects for reading versus baseline conditions. *Notes*: The intercept equals the factor level reading conditions. ***p < 0.001.

Measure		Estimate	*SE*	*df*	*t*	*p*
RR	(Intercept)	10.67	0.92	25.56	11.63	<0.001***
	Baseline	–6.57	0.44	5.12	–14.82	<0.001***
DET	(Intercept)	68.01	4.58	22.72	14.84	<0.001***
	Baseline	–37.73	3.9	5.39	–9.68	<0.001***
ADL	(Intercept)	7.16	0.78	24.59	9.17	<0.001***
	Baseline	–4.61	0.6	5.33	–7.72	<0.001***
MDL	(Intercept)	208.01	18.51	24.6	11.24	<0.001***
	Baseline	–126.84	7.42	5.08	–17.11	<0.001***
SampEn	(Intercept)	0.07	0	8.79	21.73	<0.001***
	Baseline	–0.03	0	5.9	–7.43	<0.001***

The results partially confirmed our hypothesis that reading conditions exhibit higher regularity compared to baseline conditions. Regarding RQA measures, it could be verified that reading conditions lead to higher regularity of eye movement fluctuations as compared to baseline conditions. SampEn results contradicted our prediction if interpreted as a measure of uncertainty. However, if SampEn was interpreted in terms of entropy rate (Porta et al., 2001), it rather captured the complexity of fluctuations, which were potentially related to adaptive cognitive processing.

#### Exploratory analysis: texts vs. shuffled texts vs. text grids

##### Gaze step data

In order to determine the extent to which the reading conditions differ from one another, we further set up a linear mixed-effects model for each dependent variable as a function of condition (text vs. shuffled text vs. text grid) as categorical fixed effect. Again, participant was included as random intercept.

While no significant effect of condition could be found for RR and MDL (RR: χ^2^(2) = 3.50, *p* = 0.174; MDL: χ^2^(2) = 3.36, *p* = 0.187), DET and ADL were affected by condition (DET: χ^2^(2) = 48.57, ***p < 0.001; ADL: χ^2^(2) = 35.66, ***p < 0.001). While DET was significantly lower for text grids compared to both normal texts and shuffled texts, it did not differ significantly between normal text and shuffled text. For ADL, a different pattern emerged: It significantly separated normal text from both shuffled text and text grid, but shuffled text and text grid were not distinguishable. Also, SampEn was significantly influenced by reading condition (χ^2^(2) = 114.54, ***p < 0.001). While SampEn was higher for normal text compared to both other conditions, no differences were found between shuffled text and text grid. See Table 7 for pairwise differences of the fixed factor.

**Table 7.** RQA measures (DET and ADL) and SampEn for gaze step data: Pairwise comparison of reading conditions. *Notes*: *p*-values were adjusted using the Bonferroni method for three estimates. ***p < 0.001, n.s. = not significant.

Measure	Contrast	Estimate	*SE*	*df*	*t*	*p*
DET	Normal text–shuffled text	1.39	0.98	435	1.42	0.467 n.s.
	Normal text–text grid	6.61	0.98	435	6.78	<0.001***
	Shuffled text–text grid	5.22	0.98	435	5.35	<0.001***
ADL	Normal text–shuffled text	1.43	0.36	435	3.94	<0.001***
	Normal text–text grid	2.16	0.36	435	5.98	<0.001***
	Shuffled text–text grid	0.73	0.36	435	2.03	0.128 n.s.
SampEn	Normal text–shuffled text	0.006	0.001	435	8.61	<0.001***
	Normal text–text grid	0.007	0.001	435	10.81	<0.001***
	Shuffled text–text grid	0.001	0.001	435	2.18	0.089 n.s.

Regarding gaze step data, the RQA results demonstrated that normal text tends to lead to higher regularity of eye movement fluctuations during reading compared to “impoverished” conditions, such as text grid and shuffled text. However, the different RQA measures resulted in distinctive patterns for the conditions, reflecting varying levels of sensitivity. Again, the SampEn results did not follow the pattern as one might expect from a measure of uncertainty or irregularity, but rather complexity.

##### Fixation durations

Again, linear mixed-effects models for each indicator were computed using condition (normal text, shuffled text, text grid) as categorical fixed effect and participant as random intercept.

While RR only showed a tendency (RR: χ^2^(2) = 5.71, *p* = 0.057), DET, ADL, and MDL were affected by condition (DET: χ^2^(2) = 22.60, ***p < 0.001; ADL: χ^2^(2) = 13.64, **p = 0.001; MDL: χ^2^(2) = 34.10, ***p < 0.001). As pairwise tests of fixed effects revealed (see Table 8), normal text exhibited longer ADL but shorter MDL than both other conditions. However, text grid and shuffled text conditions were not significantly different regarding ADL and MDL. DET significantly distinguished text grids from both normal and shuffled texts, with text grids showing higher DET. There was no significant effect for SampEn (χ^2^(2) = 4.01, *p* = 0.135).

**Table 8.** RQA measures for fixation duration data: Pairwise differences of conditions. *Notes*: *p*-values were adjusted using the Bonferroni method for three estimates. *p < 0.05, **p < 0.01, ***p < 0.001, n.s. = not significant.

Measure	Contrast	*Estimate*	*SE*	*df*	*t*	*p*
DET	Normal text—shuffled text	0.22	0.54	442	0.41	1.000 n.s.
	Normal text—text grid	–2.12	0.54	442	–3.94	<0.001***
	Shuffled text—text grid	–2.34	0.54	442	–4.35	<0.001***
ADL	Normal text—shuffled text	0.32	0.12	442	2.67	0.024*
	Normal text—text grid	0.43	0.12	442	3.57	0.001**
	Shuffled text—text grid	0.11	0.12	442	0.91	1.000 n.s.
MDL	Normal text—shuffled text	–38.31	7.57	442	–5.06	<0.001***
	Normal text—text grid	–39.51	7.57	442	–5.22	<0.001***
	Shuffled text—text grid	–1.21	7.57	442	–0.16	1.000 n.s.

The results once more indicate that normal reading can be distinguished from related conditions by means of RQA. Opposed to gaze step data, however, the different indicators do not all result in more regularity for normal text. Instead, task-specific patterns emerged. When applied on fixation duration data, SampEn seems noninformative in terms of separating the reading conditions.

### Discussion of experiment 1

This study aimed to test the basic assumptions of RTR, namely, that reading of text stimuli leads to higher degrees of regularity compared to baseline conditions where information—and certainly sequentially structured information—was absent. To this end, eye movements were recorded for six conditions, three baseline conditions (fixation cross, blank screen, random circles) and three reading conditions (text grid, shuffled text, normal text). We utilized RQA measures and SampEn, which can be used to capture the strength of regularity from sequential data, and tested these measures on series of gaze steps and fixation durations. Measures and the underlying data type were largely of explorative nature here in order to investigate which combination proves most sensitive for future applications of RTR to text reading.

Based on RTR, we predicted lower degrees of regularity for baseline compared to reading conditions. This was tested on gaze step data and largely supported by recurrence measures, with reading conditions exhibiting higher recurrence properties than baseline conditions. For SampEn, we assumed that higher regularity of the reading conditions would be reflected in lower SampEn values. However, the opposite pattern emerged: Reading conditions were more entropic than baseline conditions. Furthermore, we anticipated both text grids and shuffled texts to have lower degrees of regularity compared to normal text. Since the computed regularity measures were not used in this research area before, these assumptions were of an exploratory nature. Support for these predictions was mixed: Normal text showed higher recurrence properties and SampEn values compared to randomized texts and text grids for the gaze step data. For fixation data, however, DET and MDL showed opposite patterns of results (i.e., lower regularity for normal text) while ADL confirmed the expected pattern again. SampEn showed no significant effect at all. Thus, the effects observed for series of fixation durations were rather inconclusive, with recurrence measures showing positive, negative, and null effects, and null effects for entropy measures throughout.

Even though we found supporting evidence for our hypotheses, this support is weakened by the exploratory character of the analysis, as it rested on the post hoc selected combination of measures and data type. Hence, confirmatory studies are needed to strengthen this evidence.

#### Data type

Regarding the comparison of data type (gaze steps vs. fixation durations), our results clearly favored gaze step data. First, results based on series of gaze steps were generally more sensitive to our manipulations (recurrence and entropy measures yielded significant differences between condition types and among reading conditions), while RR and SampEn did not distinguish between our manipulations when calculated for fixation durations. This might partially be grounded in data size requirements: Gaze step data comprised several thousand data points per trial, whereas series of fixation durations consisted of fewer than 200 data points.

Second, the direction of effects was more in line with the predictions of RTR. Reading conditions resulted in higher degrees of regularity compared to baseline conditions when the analyses were based on gaze step data, SampEn posing an exception. When based on fixation durations, this was only true for ADL while RR and SampEn yielded null effects, and even the opposite pattern was found for DET and MDL. It might be the case that this is a result of comparatively short trial length. There are startup transients in reading tasks that span over multiple up to several hundred fixations of word reading times, leading to initially higher variability in reading task performance as would be expected for the whole task (Wallot et al., 2013, 2019). Also, different tasks produce somewhat different eye movement dynamics, and parsing such records can sometimes lead to systematically different estimates of fixation durations (Karsh & Breitenbach, 2021).

Finally, gaze step data were more versatile than fixation durations and can be used to compare qualitatively different tasks, some of which might not exhibit fixation- and saccade-like properties such as the baseline conditions that we used here.

#### Conditions and instructions

The assumptions spelled out in A1 to A4 rested on the idea of a baseline measure for eye movements, meaning absence of external information. While we tried to create three reasonable baseline conditions that were low on what can be thought of as external information, they still provide varying degrees of information to structure gaze activity. While it is probably impossible to talk about eye movements in the absence of external information in the strict sense, it would be helpful to have a general metric on information that could be applied in order to quantify the distance between the baseline and reading conditions in this regard.

Also, the chosen reading conditions offered only a first and limited insight in applying recurrence and entropy measures to the reading process. The conditions chosen did not resemble a continuous range from “information-free” contexts toward a full, naturalistic text presentation. Such an investigation would surely be interesting when focusing on variants of text-like conditions in order to clarify what different text features contribute to the reading process. However, with regard to the feasibility of this study, we had to restrict the set of conditions to some relevant contrasts for the central research question asked here, since our goal was not yet to map out the influence of different text properties on RTR, but first and foremost to establish an understanding of regularity in contexts with minimal external information compared to the processing of text-like variations and actual texts.

Furthermore, task instructions between the experimental conditions varied so that participants behaved most properly within each condition. However, this might limit the conclusions that can be drawn from the experiment, as participants’ behavior was now a function of stimuli and instruction together. The decision to use different instructions was motivated by the fact that participants can handle stimuli quite differently when not explicitly instructed. During the pilot phase of the experiment, participants were more comfortable letting their gaze wander or looking at a different part of the screen instead of staring at the displayed fixation cross for the entire 60 seconds of a trial. Similarly, participants did not necessarily engage in reading-like behavior when text grids or random text was presented, but rather let their gaze wander or even jumped back and forth in an attempt to puzzle together a meaningful text. While these spontaneous interaction patterns with different stimuli were quite fascinating, they were not pertinent to tackle the underlying research question. Still, in order to address the question of how instructions might have contributed to the observed pattern of results, we conducted a second study with a uniform instruction across conditions.

## Experiment 2

In order to address the points of varying instructions and a limited set of conditions as discussed above, we carried out an additional study. A more general but uniform instruction was used to distinguish effects driven by instructions and effects due to linguistic information conveyed by the different stimuli. Specifically, participants were told to look at the contents presented on the screen, irrespective of the particular stimulus type. Furthermore, a more differentiated set of conditions reflecting a more graduated buildup of linguistic information was chosen for this second study. At the same time, this posed a chance to corroborate the findings of the previous study and to further explore the sensitivity of measures of RTR.

### Hypotheses

**Figure 2.**
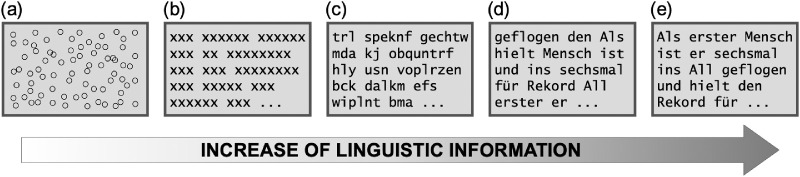
Schematic examples for the experimental conditions. Experimental conditions consisted of (a) circles, (b) text grid, (c) pseudo text, (d) randomized text, and (e) normal text.

This second study further investigated the differences captured by measures of regularity for conditions that reflect more graduated levels of external linguistic information available in a stimulus (see Figure 2). Based on the concept of RTR and the previous findings of Experiment 1, we expected strongest regularity for normal text reading. Based on our reasoning from the previous study, we expected to find more regularity in those conditions more similar to normal text. However, we have to cautiously qualify this hypothesis. Not providing participants with specific instructions of what to do might lead to different patterns of behavior. For example, eye movements differ greatly if participants read a text for comprehension, search for typos, or count the number of words in a text. While we were intuitively confident that participants would engage in normal reading behavior when presented with an actual text (this should be what skilled readers are naturally inclined to do), it was less clear how they would act in the less self-instructing conditions.

Again, regularity was operationalized by means of RQA measures (i.e., RR, DET, ADL, and MDL) as well as SampEn that were computed based on series of gaze steps. This combination of measures and data type proved most suitable to capture the different degrees of linguistic information reflected in eye movement data in Experiment 1.

### Method

#### Participants

Twenty-seven German natives with normal or corrected-to-normal vision participated in the study. They did not take part in the previous experiment and had no neuropsychological disabilities. Participants were compensated for their time with 7€ per 30 minutes and received 14€ on average. One participant had to be excluded due to problems during the calibration procedure. Three more participants dropped out of analysis due to excessive blinking artifacts in the recorded data. Thus, the final sample consisted of 23 participants (13 female) with a mean age of 26.43 years (*SD* = 4.97). See Appendix A for further information about the participants. Written informed consent was obtained from all participants prior to the experiment. As for the previous study, the method was approved by the Ethics Council of the Max Planck Society and followed the ethical principles of the Declaration of Helsinki.

#### Stimuli

All in all, Experiment 2 comprised five conditions: (a) circles, (b) text grid, (c) pseudo text, (d) shuffled text, and (e) normal text. Except for the pseudo text condition, all other conditions were part of Experiment 1 (see above for a detailed description of stimulus selection and generation). The pseudo text condition was included in order to decrease the leap between text grids and shuffled texts. While text grids preserved the general layout of a text (all letters replaced by “x” but spatial organization through spaces and lines kept intact), shuffled texts already contained semantic information on the word and topic level (randomized word order of actual newspaper articles). For the pseudo text condition, words of a text were replaced by random letter strings that do not constitute any German words and are unpronounceable for German natives.

#### Procedure

The study was carried out with the same spatial and technical setup as described above for Experiment 1. It took participants about 50 minutes to complete the experiment, including a short break halfway through the experiment. Again, participants were randomly distributed to one of two stimulus lists: Actual newspaper articles assigned to List A served as text base for conditions (b) to (e) in List B and vice versa. The experiment comprised 7 trials per condition, resulting in 35 trials in total. All trials were presented in a fully randomized order.

Participants were instructed to look at the content presented on the screen and that their gaze should remain on the screen during the entire trial. Since participants were intentionally not instructed to read in any of the conditions, there was no fixation cross preceding any of the trials. Furthermore, trial duration was set uniformly to 40 seconds. This time interval was deliberately chosen to be shorter than the average reading times obtained from Experiment 1 in order to prevent fast-reading participants from finishing before the end of the trial.

#### Data analysis

The data of the study are available here: https://osf.io/5eysw/.

##### Preprocessing

All steps regarding preprocessing were kept the same as in Experiment 1, so that a certain comparability of data and results was ensured. Due to blinks and artifacts that were detected based on the pupillometry noise algorithm (Hershman et al., 2018), data of three participants were discarded, and a total of 24 out of the remaining 805 trials (2.98%) was excluded from further data analysis. In a trial-by-trial manner, gaze steps were calculated (cf. Stephen & Mirman, 2010), and extreme values that differed more than 10 *SD* from the mean were removed. Since fixation durations turned out to be less well suited to capture the eye movement dynamics of interest in Experiment 1, these were not extracted for Experiment 2.

##### RQA and SampEn

Time series of gaze steps were subjected to RQA and SampEn analysis using the same resources as for Experiment 1, that is, the crqa package for R (Coco et al., 2021) and a custom-script for MATLAB to compute SampEn. Again, a windowed RQA was computed with a window size of 10,000 data points and a window step of 5,000 data points. Afterward, RQA measures were averaged per trial. Based on an iterative procedure, the following parameters were specified: a delay parameter *τ* = 2, an embedding parameter *D* = 4, and a threshold parameter *T* = 0.5. These parameters resulted in a mean RR of 7.30% (*SD*_RR_ = 8.25) for the whole sample. SampEn analysis was carried out with a template length *m* = 1 and a size of the tolerance region *r* = 3.0 (cf. Ramdani et al., 2009).

##### Inferential statistics

As described above, this second study investigated differences in regularity measures between five experimental conditions. Regularity was operationalized by means of the RQA measures RR, DET, ADL, and MDL, as well as SampEn. Each of these dependent variables was subjected to linear mixed-effects models using the R packages lme4 (v1.1-23) and lmerTest (3.1-2). Within the multilevel models, condition was defined as fixed factor with five levels, and a random intercept for participants was included, according to the following general form:

$$y_{mi}=y_{00}+y_{01}COND_{mi}+\upsilon_{0i}+{ɛ}_{mi},ɛ∼N\left(0,\sigma^{2}\right)$$

Here, *y*_00_ is the fixed intercept, *y*_01_*COND_mi_* is the fixed effect for condition, $\upsilon_{0i}$ is the random intercept for participants, and ε_*mi*_ is the error term.

#### Results

Table 9 provides the descriptive statistics for all dependent measures. Condition affected all regularity measures but MDL (RR: χ^2^(4) = 224.53, ***p < 0.001; DET: χ^2^(4) = 283.00, ***p < 0.001; ADL: χ^2^(4) = 54.47, ***p < 0.001; SampEn: χ^2^(4) = 289.49, ***p < 0.001; MDL: χ^2^(4) = 6.00, *p* = 0.199). For RR and ADL, values gradually increased the more linguistic information became available. Apart from two contrasts (circles vs. text grid and text grid vs. pseudo text), all other pairwise comparisons were significant. While descriptive results for ADL revealed a similar pattern, only the contrasts of normal text compared to pseudo text, text grid and circles, and random text compared to text grid and circles reached significance. SampEn did not differentiate pseudo text from text grid and circles, but it still exhibited the expected pattern of results for all other contrasts. See Table 10 for pairwise differences of the fixed factor. These findings supported the hypothesis that normal text exhibits more regularity than the other conditions. Furthermore, results mostly support the assumption that increasing availability of external linguistic information leads to increased regularity that can be meaningfully depicted by means of recurrence and entropy measures.

**Table 9.** Descriptive statistics for dependent variables

		RR		DET		ADL		MDL		SampEn	
Condition	Number of trials	*M*	*SD*	*M*	*SD*	*M*	*SD*	*M*	*SD*	*M*	*SD*
Normal text	160	11.98	10.58	26.35	19.98	3.841	1.535	41.73	57.72	0.064	0.011
Shuffled text	157	9.03	8.78	20.37	16.46	3.461	1.271	32.69	49.19	0.059	0.012
Pseudo text	154	5.83	7.07	13.57	13.73	3.195	2.002	40.45	184.20	0.051	0.013
Text grid	152	5.10	5.35	12.41	11.81	2.956	0.648	19.74	14.26	0.052	0.013
Circles	158	4.42	5.69	10.70	11.45	2.983	1.627	26.42	103.88	0.049	0.011

**Table 10.** RQA measures (RR, DET, and ADL) and SampEn: Pairwise comparisons. *Notes*: *p*-values were adjusted using the Bonferroni method for 10 estimates. *p < 0.05, **p < 0.01, ***p < 0.001, n.s. = not significant.

Measure	Contrast	Estimate	*SE*	*df*	*t*	*p*
RR	Normal text–shuffled text	3.01	0.55	762	5.46	<0.001***
	Normal text–pseudo text	6.03	0.56	762	10.849	<0.001***
	Normal text–text grid	6.78	0.56	762	12.155	<0.001***
	Normal text–circles	7.66	0.55	762	13.889	<0.001***
	Shuffled text–pseudo text	3.01	0.56	762	5.40	<0.001***
	Shuffled text–text grid	3.76	0.56	762	6.72	<0.001***
	Shuffled text–circles	4.65	0.55	762	8.38	<0.001***
	Pseudo text–text grid	0.75	0.56	762	1.33	1.000 n.s.
	Pseudo text–circles	1.63	0.56	762	2.93	0.035*
	Text grid–circles	0.89	0.56	762	1.58	1.000 n.s.

DET	Normal text–shuffled text	6.10	0.99	762	6.13	<0.001***
	Normal text–pseudo text	12.52	1.00	762	12.522	<0.001***
	Normal text–text grid	13.70	1.00	762	13.659	<0.001***
	Normal text–circles	15.87	0.99	762	15.984	<0.001***
	Shuffled text–pseudo text	6.42	1.00	762	6.39	<0.001***
	Shuffled text–text grid	7.60	1.01	762	7.55	<0.001***
	Shuffled text–circles	9.77	1.00	762	9.79	<0.001***
	Pseudo text–text grid	1.18	1.01	762	1.17	1.000 n.s.
	Pseudo text–circles	3.35	1.00	762	3.34	0.009**
	Text grid–circles	2.16	1.01	762	2.15	0.317 n.s.

ADL	Normal text–shuffled text	0.39	0.14	762	2.77	0.057 n.s.
	Normal text–pseudo text	0.63	0.14	762	4.53	<0.001***
	Normal text–text grid	0.87	0.14	762	6.24	<0.001***
	Normal text–circles	0.87	0.14	762	6.28	<0.001***
	Shuffled text–pseudo text	0.25	0.14	762	1.76	0.787 n.s.
	Shuffled text–text grid	0.49	0.14	762	3.48	0.005**
	Shuffled text–circles	0.49	0.14	762	3.48	0.005**
	Pseudo text–text grid	0.24	0.14	762	1.72	0.868 n.s.
	Pseudo text–circles	0.24	0.14	762	1.70	0.892 n.s.
	Text grid–circles	–0.00	0.14	762	–0.03	1.000 n.s.

SampEn	Normal text–shuffled text	0.005	0.001	762	5.50	<0.001***
	Normal text–pseudo text	0.013	0.001	762	13.24	<0.001***
	Normal text–text grid	0.012	0.001	762	12.94	<0.001***
	Normal text–circles	0.015	0.001	762	16.04	<0.001***
	Shuffled text–pseudo text	0.007	0.001	762	7.73	<0.001***
	Shuffled text–text grid	0.007	0.001	762	7.46	<0.001***
	Shuffled text–circles	0.010	0.001	762	10.48	<0.001***
	Pseudo text–text grid	0.000	0.001	762	–0.25	1.000 n.s.
	Pseudo text–circles	0.003	0.001	762	2.68	0.075 n.s.
	Text grid–circles	0.003	0.001	762	2.92	0.036*

As shown in Table 11, intercorrelations of regularity measures were overall high with the exception of SampEn and MDL, which showed rather moderate correlations strengths. This basically replicated findings from Experiment 1 suggesting that the utilized measures indeed capture the regularity concept well and to a similar degree.

**Table 11.** Correlation matrix for dependent variables. *Notes*: Pearson's *r* correlation coefficients. *p < 0.05, **p < 0.01, ***p < 0.001, n.s. = not significant.

Measure	RR	DET	ADL	MDL	SampEn
RR	—	0.99***	0.83***	0.50***	0.50***
DET	0.99***	—	0.81***	0.46***	0.56***
ADL	0.83***	0.81***	—	0.86***	0.24***
MDL	0.50***	0.46***	0.86***	—	0.00 n.s.
SampEn	0.50***	0.56***	0.24***	0.00 n.s.	—

### Discussion of experiment 2

This second study provided additional evidence for how measures of regularity can reliably capture varying degrees of linguistic information conveyed by visual stimuli in time-series data. Five experimental conditions were chosen, with arbitrary layouts of circles providing no linguistic context at all, and, opposed to that, short newspaper articles incorporating the maximum of linguistic information represented the extrema. Three conditions in between, text grids, pseudo texts, and texts with randomized word order, comprised increasing levels thereof. Again, recurrence and entropy measures were used to capture the strength of regularity based on series of gaze steps.

We hypothesized that regularity measures should be highest for normal text and lower for the other conditions. This prediction was borne out by the observed results. Furthermore, we more cautiously presumed that increasing linguistic information could be reflected by increasing regularity measures. Also, this assumption was mostly supported by the results. Since these results were observed when task instructions were kept constant across conditions, we can be confident in the validity of the findings of Experiment 1. At the same time, however, the uniform instructions impede a further interpretation of significant effects (or the lack thereof) for some of the conditions with intermediate linguistic information (i.e., with regard to differences between shuffled texts, pseudo texts, and text grids).

## General discussion and outlook

The central aim of the present article was to test a fundamental assumption of RTR. That is, with enhancing degrees of external (linguistic) information, the regularity of dynamical measures that reflect processing during reading increases. To prove this, we used measures that capture the regularity enclosed in time series, here specifically measures of recurrence and entropy. These measures were applied to eye movements that we recorded for contexts in which linguistic information was absent, increasingly text-like conditions, and the presentation of actual texts. Findings across two experiments showed that regularity measures distinguished successfully between text reading and conditions with varying degrees of linguistic information. However, some specific patterns of results emerged for the different regularity measures that need to be further discussed. In particular, SampEn did not behave in a way that warrants a plain interpretation in terms of regularity. Furthermore, we would like to discuss the limitations of the studies reported here and provide an outlook for future research.

### Measures

Conceptually, recurrence and entropy measures imply a fairly straightforward interpretation: Higher regularity in a time series of eye movements should be reflected in higher values for RQA measures and lower values for SampEn. And indeed, the first part of this notion was supported by our results: Recurrence measures consistently indicated higher regularity for reading conditions compared to baseline conditions and, for gaze step data, also higher regularity the more similar stimuli were to normal texts. However, results for SampEn opposed this tenet. While SampEn did prove to be a sensitive measure for regularity, its effects seemed to contradict the concept of RTR.

A possible explanation for this might be that SampEn is, strictly speaking, not a classical entropy measure. As pointed out in the Introduction, the calculation of SampEn is based on how well smaller templates in a time series extend to larger ones. Hence, it might be more similar to measures of entropy rate (Porta et al., 2001) than to entropy measures per se. As entropy rate captures complexity of data (i.e., the presence of multiple systematic patterns in a time series), it rather captures complexity of a signal and indexes adaptive cognitive processing but not irregularity.

What does this imply? One of the exploratory aims of the current study was to use different potentially suitable measures to capture RTR and investigate which of these prove to be sensitive. While SampEn did turn out to capture the dynamics of interest, the direction of effects is not easily reconcilable with the notion of RTR. If SampEn would indeed be interpreted as a complexity measure, it might capture an aspect of skilled reading that is not (yet) incorporated into the concept of RTR, namely, adaptive flexibility. As outlined above, RTR focusses on the stability of reading behavior over time that is expected to arise from skilled reading. However, skill behavior also has an adaptive component that is not reflected within stability, that is, skill execution of behavior also entails quick and successful adaption to changes in the situation (Riley & Turvey, 2002; Ward et al., 2018).

Interpreted this way, SampEn as a complexity measure might rather capture this adaptability facet of skill. Consequently, skilled reading would be marked by high stability of the process but, at the same time, by high adaptive flexibility. This notion would also be in line with findings that multifractal measures that capture complexity of behavior (e.g., Ihlen & Vereijken, 2010; Kelty-Stephen & Wallot, 2017) are also increased in high-skilled readers (Wallot et al., 2014). However, this train of thought warrants a theoretical expansion of the RTR concept that has yet to be conceptualized.

### Limitations

The conclusions that can be drawn from the current studies are limited by several factors. First of all, the assumptions spelled out in A1 to A4 rest on the idea of a baseline measure for eye movements as such, that is, the absence of external information. While we tried to create three reasonable baseline conditions low on what can be thought of as external information, they do still provide varying degrees of information to structure gaze activity. While it is probably impossible to talk about eye movements in the absence of external information in a strict sense, it would be helpful to have a general metric on information that could be used to quantify the distance of the baseline conditions and reading conditions in this regard. Also, while we find supporting evidence for our hypotheses, this support is weakened by the exploratory character of the analysis, as it rests on the post hoc selected combination of measures and data type. Hence, confirmatory studies are needed to strengthen this evidence.

Here, it also has to be mentioned that the current approach on evaluating regularity metrics rests on individual evaluations in separate univariate analyses. While this serves our goal of identifying which of these metrics are suitable and sensitive operationalizations of RTR, a multivariate combination of these measures might yield further insights or even better separability of conditions.

Furthermore, the results based on gaze step and fixation durations of the first experiment are not fully comparable. Some of the metrics employed here gain in reliability with increasing length of a time series. Accordingly, results based on gaze steps might merely be more sensitive to the experimental manipulations by virtue of greater time series length compared to fixation-based results.

Finally, RTR was formulated for the application to reading tasks (Wallot 2014, 2016), especially to connected text reading. However, text stimuli of the current study consisted of only relatively short newspaper articles that tend to work differently than longer connected texts (Wallot et al., 2013, 2019). Accordingly, future studies need to validate the current findings on longer text stimuli.

### Outlook

In the current studies, we introduced RTR as a means to capture the process of connected text reading. Our results support that RTR adequately captures the difference between nonreading and reading conditions, as well as show evidence for the assumption that sequential information inherent in text reading leads to stronger regularity of reading process measures. Furthermore, our results suggest that recurrence measures and SampEn are well-suited measures to capture RTR. Moreover, when using eye movements, gaze step data seem to be the better basis for such analyses compared to series of fixation durations.

However, reading ultimately pursues the goal of gaining information, that is, comprehending a text. Thus, the motivation for RTR originates in text comprehension research and the questions of whether and how comprehension can be predicted by means of process measures of reading across tasks (Teng et al., 2016) and languages (Frost, 2012). On the one hand, various measures of the reading process such as word or sentence reading times, fixation durations, or the number of regressive eye movements have been shown to vary with local or global text difficulty (e.g., Just & Carpenter, 1980; Rayner et al., 2006). Using such measures to predict comprehension, on the other hand, has been far from trivial and did not always succeed (LeVasseur et al., 2006, 2008).

Some studies that utilized regularity metrics had some success in predicting comprehension from reading times and eye movements (Wallot et al., 2014, 2015). The current article was based on this work. But also other recent studies have successfully predicted comprehension using the notion of coupling between text features and perceptual-cognitive processing. For instance, Mills and colleagues (2017) showed that reading times and cognitive coupling, operationalized as regression of reading times and text complexity, were positive predictors of participants’ reading comprehension. Moreover, they demonstrated that decoupling measured in instances of mind-wandering resulted in worse text comprehension. Moreover, Southwell and colleagues (2020) showed that comprehension scores can be successfully predicted from reading times and classical eye movement measures. However, it remains unclear why the same measures yielded null effects in other studies (Wallot et al., 2015) or related reading speed components during self-paced reading (LeVasseur et al., 2006, 2008; Wallot et al., 2014). Potentially, this might be traced back to differences in modeling and sample size, but also to how comprehension was assessed, and the parameter settings applied to define reading times or extract fixations.

Conceptually, we do see a potential advantage for RTR-based measures because they do not depend on defining text properties whose effects might not be independent of task and language. However, whether RTR offers better metrics to predict reading comprehension from process data is an empirical question that will have to be addressed in future studies, investigating the relation between the reading process and comprehension, directly comparing the different successful approaches on the same data sets but also across important variations such as different types of reading tasks and writing systems.

## Appendix A: Participant information

**Table:**

Experiment	Participant	List	Sex	Age (years)	Languages	Education	Vision problems	Vision aid	Reading (hours per week)
Experiment 1	1	A	male	24	3	2	no	none	45
	2	B	female	21	3	1	yes	contacts	20
	3	B	female	21	3	1	no	none	10
	4	A	female	29	4	2	no	glasses	10
	6	B	female	28	3	2	no	none	24
	7	A	male	37	4	1	yes	glasses	40
	9	A	female	25	5	2	no	none	4
	10	B	male	21	3	1	no	none	6
	11	A	female	23	3	1	no	none	14
	15	A	female	28	2	1	yes	contacts	12
	16	A	female	57	2	3	no	glasses	15
	17	A	female	25	3	2	no	glasses	35
	18	B	male	28	3	2	no	none	18
	19	B	male	23	2	2	no	none	10
	20	B	female	34	2	1	no	none	30
	21	A	male	21	2	2	no	none	20
	23	A	male	51	2	3	yes	glasses	15
	24	B	female	24	4	1	no	none	3
	25	B	female	21	3	1	no	none	21
	26	B	female	22	3	1	no	none	14
	27	B	female	24	4	1	no	none	10
	28	B	female	21	4	1	yes	contacts	12

Experiment 2	2	B	male	29	4	2	yes	contacts	30
	3	A	male	24	4	2	no	none	8
	5	A	female	24	2	1	yes	glasses	60
	6	B	female	20	5	1	no	none	12
	7	A	female	21	3	1	no	none	14
	8	B	female	23	4	1	no	none	18
	10	B	female	29	4	2	yes	contacts	14
	11	A	female	29	3	2	yes	glasses	14
	12	B	male	22	3	1	no	none	8
	13	A	female	27	4	1	yes	contacts	8
	14	B	male	31	4	2	yes	glasses	20
	15	A	female	23	3	2	no	none	10
	16	B	female	23	2	1	yes	glasses	25
	17	A	female	38	3	3	no	none	14
	18	B	male	24	2	2	no	none	12
	19	A	male	24	4	1	yes	glasses	5
	20	B	female	22	5	1	no	none	21
	22	B	female	31	4	4	no	none	10
	24	B	female	30	3	2	no	none	15
	26	B	male	25	4	1	yes	contacts	20
	27	A	male	39	3	3	no	none	30
	28	B	male	24	2	1	yes	glasses	14
	29	A	male	26	4	2	yes	glasses	14

*Notes*: All sociodemographic variables were collected through a short survey after completion of the experiment. Reported values were self-indications by the participants. “List” refers to the assigned list of texts; “Languages” refers to the number of languages participants could fluently speak and read; “Education” indicates participants' highest education level (1: university entrance degree, 2: bachelor's degree or equivalent, 3: master's degree or equivalent, 4: others).

## Appendix B: Text characteristics.

**Table:**

													Type frequency		Log type frequency		Annotated type frequency		Log annotated type frequency	
List	Title	Section	Date of publication	Words	Sentences	Clauses	Syllables	Graphemes	Words per sentence	Words per clause	Sylla-bles per word	Graph-emes per word	*M*	*SD*	*M*	*SD*	*M*	*SD*	*M*	*SD*

A	Berufstätigkeit: Mütter arbeiten länger	Economics	Jan. 23, 2018	180	10	16	374	1,039	18.00	11.25	2.08	5.77	31,3759.03	697,674.17	4.18	1.53	285,918.10	645,816.57	4.06	1.54
	Bestseller: Überraschungs-erfolg für Lundes „Bienen“	Feuilleton	Jan. 4, 2018	195	10	14	375	1,109	19.50	13.93	1.92	5.69	492,106.81	949,559.24	3.98	1.96	439,482.36	877,877.76	3.87	1.96
	Geldautomaten: Schäden steigen auf 2,2 Millionen Euro	Finances	Jan. 15, 2018	194	11	17	447	1,192	17.64	11.41	2.30	6.14	304,975.16	642,842.97	3.79	1.92	266,763.17	569,289.14	3.66	1.92
	Soziales: Gegen Pflichtbesuche in KZ-Gedenkstätten	Politics	Jan. 9, 2018	177	11	22	406	1,186	16.09	8.05	2.29	6.70	448,690.65	888,615.04	4.03	1.85	373,845.26	764,229.37	3.81	1.93
	Gesundheit: TV-Köche missachten ständig die Hygiene	Science	Jan. 22, 2018	157	9	19	343	1,008	17.44	8.26	2.18	6.42	349,691.41	747,197.27	3.80	1.89	289,416.01	637,628.42	3.72	1.85
	Mädchen sind „mutig“? Falsch! Eltern empören sich über eine skurrile Schulaufgabe	Society	Jan. 27, 2018	201	15	29	397	1,174	13.40	6.93	1.98	5.84	359,319.15	720,286.47	4.27	1.54	298,636.54	625,302.44	4.11	1.57
	Fußball: Goretzkas Wechsel zum FC Bayern perfekt	Sports	Jan. 20, 2018	189	12	22	389	1,054	15.75	8.59	2.06	5.58	373,833.61	773,071.25	3.93	2.03	285,300.86	632,720.09	3.74	2.02

	Overall List A			184.71	11.14	19.86	390.14	1,108.86	16.83	9.77	2.12	6.02	377,482.26	69,092.81	4.00	0.18	319,908.90	62,937.58	3.85	0.17

B	Niki-Verkauf: Bundesregierung pocht weiter auf Wettbewerb	Economics	Jan. 3, 2018	197	14	20	443	1,234	14.07	9.85	2.25	6.26	447,430.25	877,364.55	3.98	1.96	396,870.81	796,134.97	3.91	1.94
	Berliner Museums-insel: James-Simon-Galerie wieder im Zeitplan	Feuilleton	Jan. 12, 2018	162	10	13	369	1,016	16.20	12.46	2.28	6.27	421,329.65	844,266.78	3.87	1.93	375,649.46	782,299.27	3.75	1.92
	Übernahmen: Milliardärsfamilie sucht Geldgeber	Finances	Jan. 3, 2018	197	12	23	435	1,196	16.42	8.57	2.21	6.07	412,412.61	882,916.58	3.68	2.05	368,480.32	803,229.19	3.52	2.06
	Russland: Beschützer des Bargelds	Politics	Jan. 13, 2018	187	9	24	393	1,160	20.78	7.79	2.10	6.20	442,794.23	898,932.76	4.08	1.76	342,680.51	745,545.09	3.94	1.74
	Gesundheit: Zahn-pasta könnte gegen Malaria helfen	Science	Jan. 19, 2018	179	10	14	366	1,080	17.90	12.79	2.04	6.03	451,792.01	862,434.12	4.01	1.93	371,003.02	771,694.36	3.82	1.91
	Belgien ist in der Silvesternacht geschrumpft: Grenze zu Nieder-landen wurde zu Neujahr korrigiert	Society	Jan. 2, 2018	189	14	19	389	1,184	13.50	9.95	2.06	6.26	378,199.22	765,992.15	4.09	1.72	328,029.85	671,520.36	3.90	1.79
	Deutschland enttäuscht bei Handball-EM: Nationalteam nur mit Remis gegen Mazedonien	Sports	Jan. 18, 2018	158	7	20	353	1,007	22.57	7.90	2.23	6.37	448,362.60	874,239.04	3.88	2.00	399,975.03	805,889.56	3.76	1.97

	Overall List B			181.29	10.86	19.00	392.57	1,125.29	17.35	9.90	2.17	6.21	428,902.94	26,891.67	3.94	0.14	368,955.57	26,318.52	3.80	0.14
